# Micro-climatic variations across Malawi have a greater influence on contamination of maize with aflatoxins than with fumonisins

**DOI:** 10.1007/s12550-022-00471-1

**Published:** 2022-11-29

**Authors:** Justin Temwani Ng’ambi, Joseph Atehnkeng, Maurice Monjerezi, Cosmo Ngongondo, Ephraim Vunain, Connel Ching’anda, Alejandro Ortega-Beltran, Peter J. Cotty, Limbikani Matumba, R. Bandyopadhyay

**Affiliations:** 1 Faculty of Science, University of Malawi, P.O. Box 280, Zomba, Malawi; 2 International Institute of Tropical Agriculture (IITA), Route Kavumu, Km 18, Bifurcation, Birava, Site UCB, PO Box 1222, Bukavu, Democratic Republic of the Congo; 3 School of Plant Sciences, University of Arizona, Tucson, AZ USA; 4 International Institute of Tropical Agriculture (IITA), Ibadan, Nigeria; 5 College of Food Science and Engineering, Ocean University of China, Qingdao, China; 6 USDA-ARS, Tucson, AZ USA; 7 Food Technology and Nutrition Group, Lilongwe University of Agriculture and Natural Resources (LUANAR), Natural Resources College, P.O. Box 143, Lilongwe, Malawi; 8 MYTOX-SOUTH International Thematic Network, Ghent University, Ghent, Belgium

**Keywords:** Aflatoxins, Fumonisins, Maize, Agroecological zones, Malawi

## Abstract

This study reports levels of aflatoxin and fumonisin in maize samples (*n* = 1294) from all agroecological zones (AEZs) in Malawi. Most maize samples (> 75%) were contaminated with aflatoxins and 45% with fumonisins, which co-occurred in 38% of the samples. Total aflatoxins varied across the AEZs, according to mean annual temperature (*P* < 0.05) of the AEZs. Samples from the lower Shire AEZ (median = 20.8 µg/kg) had higher levels of aflatoxins (*P* < 0.05) than those from the other AEZs (median = 3.0 µg/kg). Additionally, the majority (75%) of the positive samples from the lower Shire AEZ had aflatoxin levels exceeding the EU regulatory limit (4 µg/kg), whereas 25%, 37%, and 39% of positive samples exceeded the threshold in the mid-elevation, Lake Shore and upper and middle Shire, and highlands AEZs, respectively. The lower Shire AEZ is characterised by higher mean temperatures throughout the year and low erratic rainfall. However, total fumonisins did not show significant variation across AEZs, but all positive samples exceeded 150 µg/kg, required for tolerable daily intake of 1.0 µg/kg body weight per day, established by the European Food Safety Authority Panel on Contaminants in the Food Chain. Therefore, results of this study suggest that contamination of maize with aflatoxin responds to micro-climate more than with fumonisins. In addition, the data will be useful to public health policy-makers and stakeholders to articulate and implement monitoring and mitigation programs.

**Supplementary Information:**

The online version contains supplementary material available at 10.1007/s12550-022-00471-1.

## Introduction

In sub-Saharan Africa, maize (*Zea mays* L*.*) is the primary staple crop accounting for about 73% of the total food demand (Shiferaw et al. 2011; Nago et al. 1997). In Malawi, food security is generally equated to sufficiency of maize supply, which accounts for more than 60% of the total food production (Government of Malawi 2012), more than 60% of energy, 67% of iron, 65% of zinc, and 70% of riboflavin consumption (Ecker and Qaim 2011). However, maize is prone to pre- and post-harvest colonisation by fungi that, under favourable conditions, produce mycotoxins (Shephard 2003). Mycotoxins, such as aflatoxins and fumonisins, are of concern because they are inimical to human and animal health, food security, and trade (Silva et al. 2018; Hendrickse 1997; Wild 2007; Williams et al. 2004; Wu 2015). Aflatoxin B1 (AFB_1_) is known to cause liver cancer (Stoloff 1983; IARC 2012; Wu 2015), growth suppression, immune system modulation, and malnutrition in humans (Kimanya et al. 2015), depending on nature of exposure (chronic or acute). In some cases, acute aflatoxicosis may lead to death (Lewis et al. 2005; Probst et al. 2007; Yard et al. 2013). As a result of its ability to suppress the immune system, AFB_1_ exposure is also associated with increased severity of diseases such as HIV/AIDS (Jiang et al. 2008; Williams et al. 2010; Jolly 2014), malaria (Allen et al. 1992), and tuberculosis (Williams et al. 2004; Keenan et al. 2011). On the other hand, fumonisins have been linked to cases of oesophageal carcinoma (Rheeder et al. 1992; Sun et al. 2007) and neural tube defects (NTDs) in populations exposed to contaminated maize (Marasas et al. 2004).

In Malawi, periodical surveys have reported high levels of contamination and co-occurrences of aflatoxin and fumonisins in maize (Chipinga 2014; Matumba et al. 2014a, b, c; Mwalwayo and Thole 2016), which is persistent over time (Magamba et al. 2017; Seetha et al. 2018). Although there have been no reports of outbreaks of severe mycotoxicosis in Malawi, there are other reported public health challenges with an epidemiological link to chronic dietary intake of aflatoxins and fumonisins. Notably, Malawi has one of the highest oesophageal cancer prevalence rates in the World (Banda et al. 2001; Nahvijou et al. 2019; Mlombe et al. 2009; Msyamboza et al. 2012; Ferlay et al. 2015; Schaafsma et al. 2015; Murphy et al. 2017; Arnold et al. 2020), which have been epidemiologically linked to high consumption of fumonisin-contaminated maize (Crofts 2008; Kachala 2010; Mlombe et al. 2015; Chetwood et al. 2018). In addition, in Malawi, prevalence of stunting, height for age (% of children under 5), is estimated at 37% (NSO 2017), which is significantly higher than the global average of 21% and the African prevalence of 29% (WHO 2021). Further, Seetha et al. (2018) reported high aflatoxin-lysine adducts in blood serum of the Malawian rural population, which correlated with high consumption of contaminated maize and groundnuts.

Sufficient and reliable data on contamination of the main staples with aflatoxins and fumonisins are necessary to devise anticipatory actions to forestall mycotoxicosis and avert large impacts to food security and safety at national level (Lewis et al. 2005; Probst et al. 2007; Yard et al. 2013; Battilani et al. 2016). In addition, variation of contamination of maize with aflatoxins and fumonisins across AEZs in Malawi, with micro-climate variations, is underexplored on a national scale. Such information would be useful to public health policy-makers and other stakeholders to target monitoring and intervention programs for subsistence populations living in extreme micro-climatic zones (Matumba et al. 2014c). To this end, this study reports on levels of aflatoxin and fumonisin in maize across all AEZs in Malawi, thereby establishing a nationwide geographical pattern and provides an extensive characterisation of the extent of variability of levels of contamination and co-occurrence of the two mycotoxins in maize under differing climatic patterns.

## Materials and methods

### Study area

**Fig. 1:**
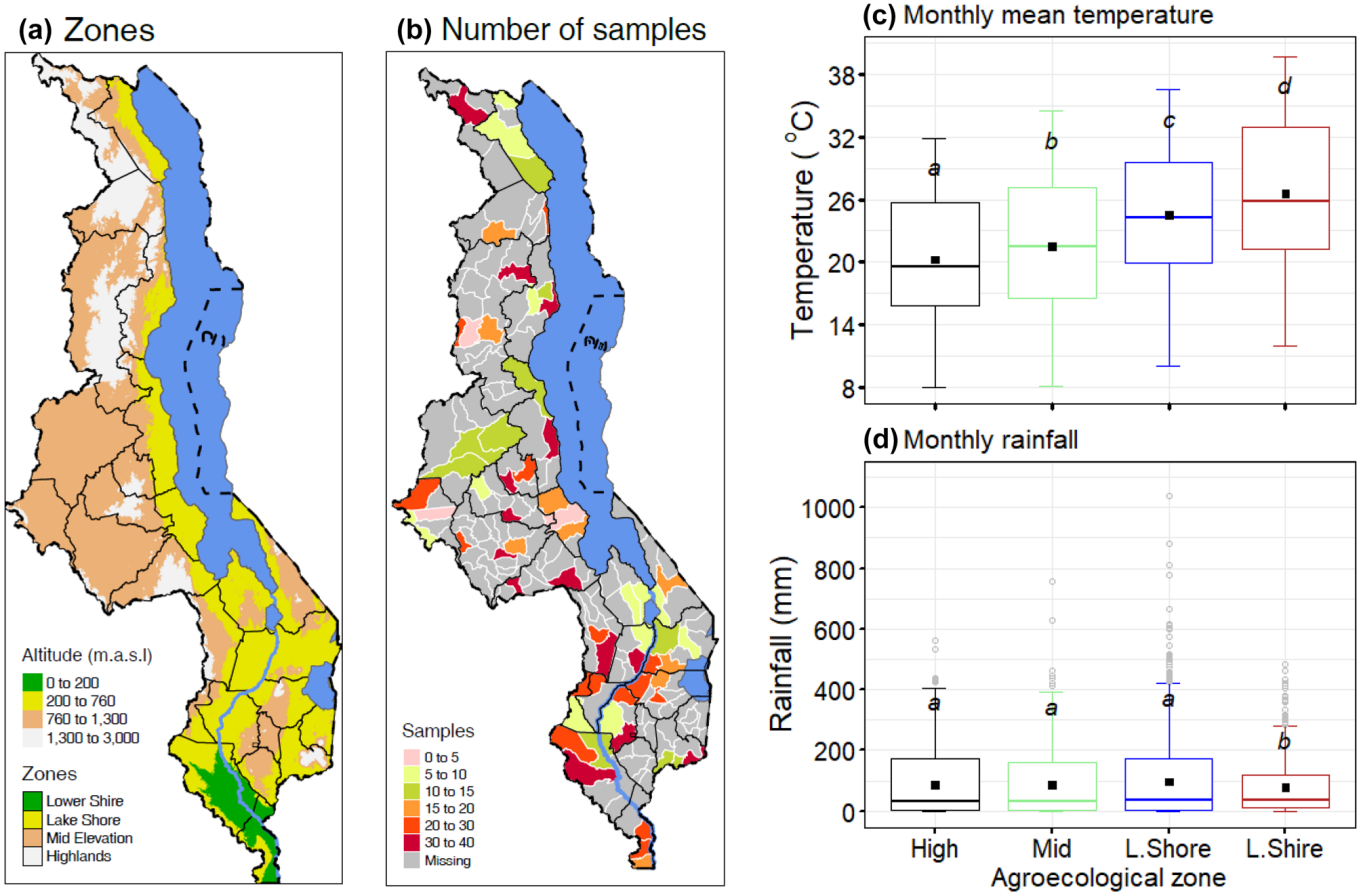
Map of Malawi showing agroecological zones and distribution of maize samples and boxplot of temperature and rainfall. **a** Map with location of the agroecological zones lower Shire (L.Shire, altitude below 200 m.a.s.l), Lake Shore and middle and upper Shire (L.Shore, > 200 to 760 m.a.s.l), mid-elevation (mid, > 760 to 1300 m.a.s.l), and highlands (high, > 1300 m.a.s.l). **b** Distribution of maize samples in each district depending on agroecological zones (elevation). **c** Monthly average temperatures in agroecological zones. **d** Monthly average rainfall in agroecological zones

Malawi may be divided into AEZs, largely according to elevation, as lower Shire (altitude below 200 m.a.s.l), Lake Shore, middle and upper Shire (> 200 to 760 m.a.s.l), mid-elevation (> 760 to 1300 m.a.s.l), and highlands (> 1300 m.a.s.l) (Matumba et al. 2014c) (Fig. 1a). The spatial variation of climate parameters (temperature, humidity, and rainfall) depends on elevation (Ravaderkar et al. 2012). Therefore, the AEZs also represent spatial climatic zonation of the country (Ngongondo et al. 2011). Maize samples were collected from a wide selection of Extension Planning Areas (EPAs) across all AEZs in Malawi to provide a wide geographical coverage (Fig. 1b).

Annual rainfall varies from 700 mm in the low-lying areas of the lower Shire to 2500 mm in the highlands (BGS 2004). The lower Shire is characterised by higher average monthly temperatures (21–30 °C) and lower monthly rainfall than the other AEZs (Fig. 1c, d). All the AEZs have a tropical wet and dry “savanna” climate (Met Malawi 2006), characterised by a distinct rainy season between November and April (Met Malawi 2006). Monthly average temperatures are around 16–23 °C in the highlands, 17–25 °C in the mid-elevation, and 21–28 °C in the Lake Shore, middle and upper Shire valley AEZs (Met Malawi 2006). As reference evapotranspiration and relative humidity levels are tied to temperature and precipitation, they show similar spatial trends.

### Collection of samples of maize grains

A total of 1294 samples of maize grains, intended for human consumption, were collected from rural households and markets from August to October 2016. This study targeted maize grains harvest from the November 2015 to April 2016 growing season. From each respondent, a sample (approximately 1 kg) was collected through donations, following introduction and discussion of the project. For grains stored in bags, the maize grains were sampled from different parts of the bag using a cylindrical bag sampler (approximately 1 m long with a 40 mm external diameter), and the aggregate sample was mixed to make a 1-kg representative sample (Whitaker 2006). The bag sampler was pushed into a bag twice through both tips of the hand-sewn end (to minimise damaging the bag) and diagonally into the bag placed horizontally to the ground. To improve sample homogeneity, the bag sampler was pushed into the bag with the intake aperture facing down, turned 180°, agitated to fill the bag sampler, and then withdrawn from the bag. Otherwise, the respondents donated approximately 1 kg of grain stored in other types of containers such as baskets. The samples were packed in thin nylon bags, placed in woven polypropylene sacks (50 kg), and then transported to and stacked in a storeroom (at room temperature) at Chitedze Research Station, Lilongwe, Malawi. The samples were analysed for total aflatoxins and fumonisin within 3 days.

### Determination of total aflatoxins and total fumonisins

Levels of total aflatoxins (AFB_1_ + AFB_2_ + AFG_1_ + AFG_2_) and total fumonisins (FB_1_ + FB_2_) in the samples of maize grains were determined using Reveal^®^ Q^+^ immunoassay kits, according to the manufacturer’s directions (Neogen^®^ Corporation, Lansing, MI, USA). Reveal^®^ Q^+^ is a single-step lateral flow immunochromatographic assay based on a competitive immunoassay format intended for quantitative testing of aflatoxin and fumonisin in grain and grain products. The kits are validated methods, have good cross-reactivity profile and recovery rate (Le et al. 2019), and are approved by the Grain Inspection, Packers, and Stockyards Administration (GIPSA) of the US Department of Agriculture (FGIS 2016, 2022).

Briefly, the samples (1 kg) were thoroughly mixed, and 500 g of which was ground using a blender (Vitamix 300 professional blender, USA), until 75% of its particles could pass through a size 20-mesh sieve. The ground samples were stored in plastic bags in a cool, dry place until time for analysis. For analysis, 20 g of each finely ground sample material was weighed into a 250-ml round-based flask using a top-loading pan analytical balance (Mettler Toledo MS104TS/00, Germany). Then, 100 ml of 65% ethanol (35% double distilled water, v/v) was added to the flask. Aflatoxins and fumonisins were extracted by shaking the mixture using a rotary shaker (GFL 3017; GFL; Burgwedel, Germany) for 3 min. The mixture was then filtered through Whatman No. 1 filter paper, and both aflatoxin and fumonisin assays were performed on the filtrate of the 65% ethanol extract. One hundred microliters of each sample extract was transferred into a sample dilution cup. For total aflatoxins analysis, 500 µl of aflatoxin sample diluent was then added to the sample dilution cup and homogenized. For fumonisins determination, 200 µl of fumonisins sample diluent was added to the 100 µl of each sample extract and homogenised in the sample dilution cup. Then 100 µl of the diluted sample extract was transferred into a new clear sample cup. The Reveal ^®^Q^+^ Kits test strips for aflatoxins and fumonisins were placed into the respective sample extracts for 6 min to develop. The developed strips were removed from the sample cups and inserted into a Reveal AccuScan Gold Reader System (AccuScan Gold Reader 9595, Neogen^®^ Corporation, Lansing, MI, USA) for quantitation of aflatoxin or fumonisin content of the sample.

The Reveal ^®^Q + Kits for aflatoxin and fumonisins had detection and quantification limits of 2–150 µg/kg and 0.3–6 mg/kg for the total aflatoxins and fumonisins, respectively. Samples whose aflatoxin and fumonisin content was above 100 µg/kg and 5 mg/kg, respectively, were diluted and reanalysed by repeating the test procedure. The results obtained from aflatoxins and fumonisins analysis were recorded in µg/kg and mg/kg, respectively. Each grain sample was treated independently and analysed in duplicate using separate sample portions. The AccuScan Gold Reader was calibrated by using standard samples provided by the manufacturer (Neogen^®^ Corporation, Lansing, MI, USA) before each reading was taken. Laboratory performance for the analytical method was assured by participation in proficiency test in global mycotoxin proficiency testing (PT) scheme operated by the Office of the Texas State Chemist (OTSC)–Texas A&M AgriLife Research (OTSC 2022). For all rounds, *z*-scores have consistently been between − 2 and + 2, which is considered satisfactory. The OTSC is ISO/IEC 17,025:2017 accredited for aflatoxin and fumonisin testing using the ELISA test kits and ISO/IEC 17,043:2010 accredited for conducting proficiency testing (OTSC 2022).

### Data analysis

All statistical analyses were performed using the R: A language and environment for statistical computing version 4.1.0 (R Core Team 2021). The non-parametric paired Wilcoxon test was used to assess the impact of geographical variation (AEZ) on levels of fumonisins and aflatoxins by pooling data at Agricultural Extension and Planning Area (EPA) level in each AEZ, using *stat_compare_means* in *ggpubr* package. Relationships between levels of aflatoxins and fumonisins in maize grain and mean annual temperature were assessed using the non-parametric Spearman’s rank test (Spearman 2010). Non-parametric techniques were used because of the non-Gaussian distribution of the datasets. Thematic maps showing spatial variation of levels of fumonisins and aflatoxins at EPA level were generated using the *tmap* package, and all boxplots were implemented in *ggplot2* package.

## Results and discussion

### Levels of total aflatoxins and fumonisins in maize grains

**Fig. 2:**
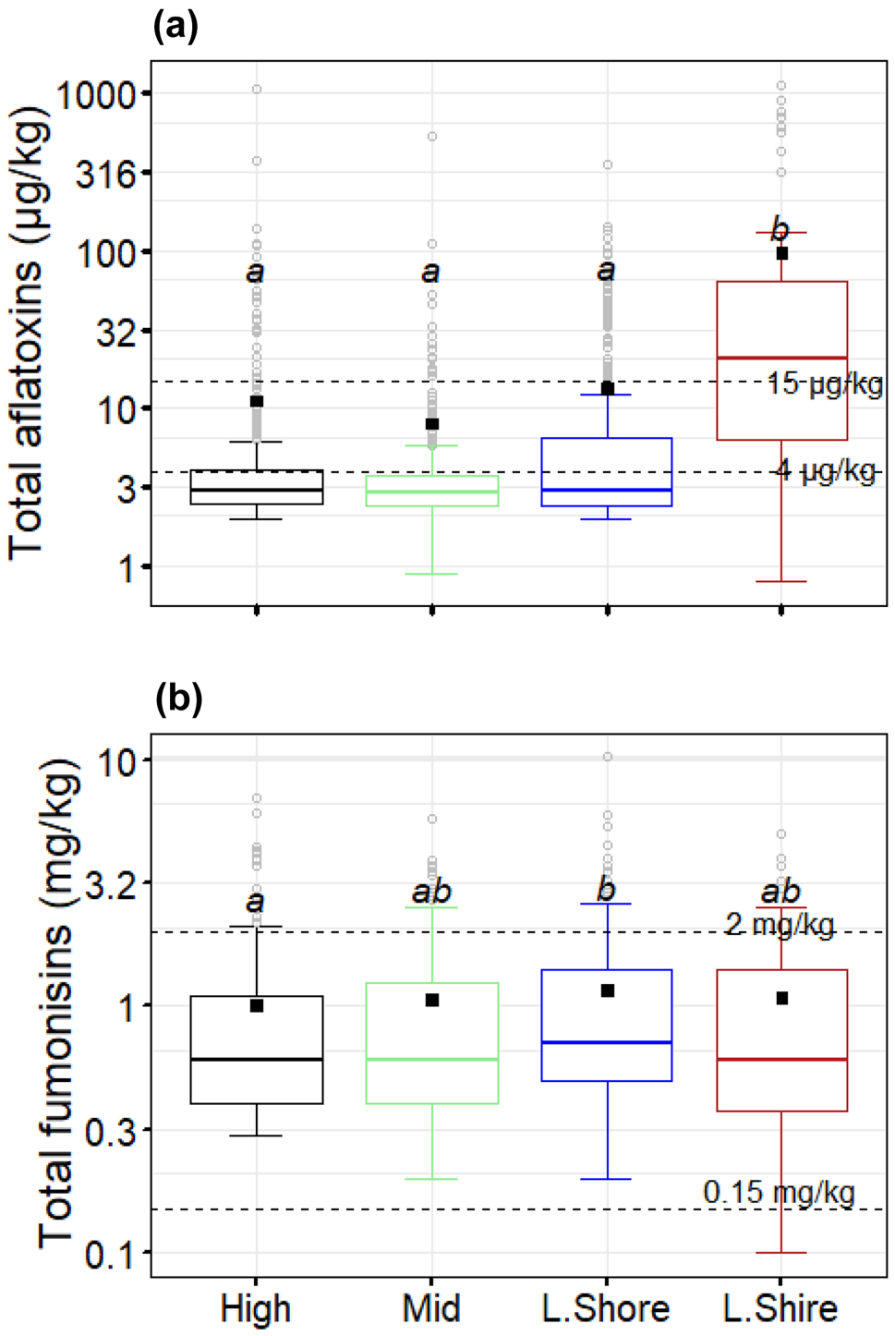
Distribution of **a** total aflatoxins and **b** total fumonisins in maize samples grouped by agro-ecological zones of Malawi. The horizontal dashed lines indicate guideline values (aflatoxins, 4 and 15 µg/kg; fumonisins, 0.15 and 2 mg/kg). Agroecological zones with the same letter have insignificant differences (*P* = 0.05). The small black square in the boxplots indicates a mean for each agroecological zone. The dots, error bars, and upper and lower ends of the box represent outliers, spread, and first and third quartiles, respectively. Agroecological zones: highlands (high); mid-elevation (mid); Lake Shore and upper and middle Shire valley (L.Shore); lower Shire valley (L.Shire)

A detailed compilation of results for levels of total aflatoxins and fumonisins in samples of maize grains is provided in supplementary Table s1 and supplementary Table s2, respectively. Total aflatoxins were detected in 78.8% of all maize samples, with a distribution of occurrences across the AEZs of 76.9%, 78.9%, 83.3%, and 76.4% for the mid-elevation, lower Shire, highlands, and Lake Shore and upper and middle Shire, respectively. On an average, maize samples collected from lower Shire AEZ had relatively higher levels of aflatoxins (*P* < 0.05) than maize samples from the other AEZs (Fig. 2a). For samples of maize grain from lower Shire, aflatoxin levels ranged from 0.8 to 1122 µg/kg (Nsanje District), with an average of 100 ± 23.1 µg/kg. For the highlands, total aflatoxin levels ranged from 2 to 1072 µg/kg (Mzuzu), with an average of 11.9 ± 3.8 µg/kg. In the Lake Shore and upper and middle Shire AEZs, total aflatoxins ranged from 2 to 358 µg/kg (Salima District, mean = 13.3 ± 1.7 µg/kg; Table s1), whereas for the mid-elevation, values were in the range of 0.9 to 540 µg/kg (Rumphi District, mean = 8.5 ± 2.2 µg/kg; Table s1). In contrast to total aflatoxins, levels of total fumonisins in maize did not show a broad variation across the four AEZs (Fig. 2b). Fumonisins were detected in 48.6%, 47.8%, 38.6%, and 44.7% of the samples collected from the highlands, Lake Shore and Upper and middle Shire, lower Shire, and mid-elevation AEZs, respectively. Levels of fumonisins ranged from 0.3 to 7.0 mg/kg (mean = 1.1 ± 0.1 mg/kg), 0.2 to 10.3 mg/kg (mean = 1.1 ± 0.1 mg/kg), 0.1 to 5.0 mg/kg (mean = 1.05 ± 0.16 mg/kg), and 0.2 to 5.8 mg/kg (mean = 1.08 ± 0.08 mg/kg), in the positive samples from the highlands, Lake Shore and upper and middle Shire, lower Shire, and mid-elevation AEZs, respectively (Fig. 2b).

**Fig. 3:**
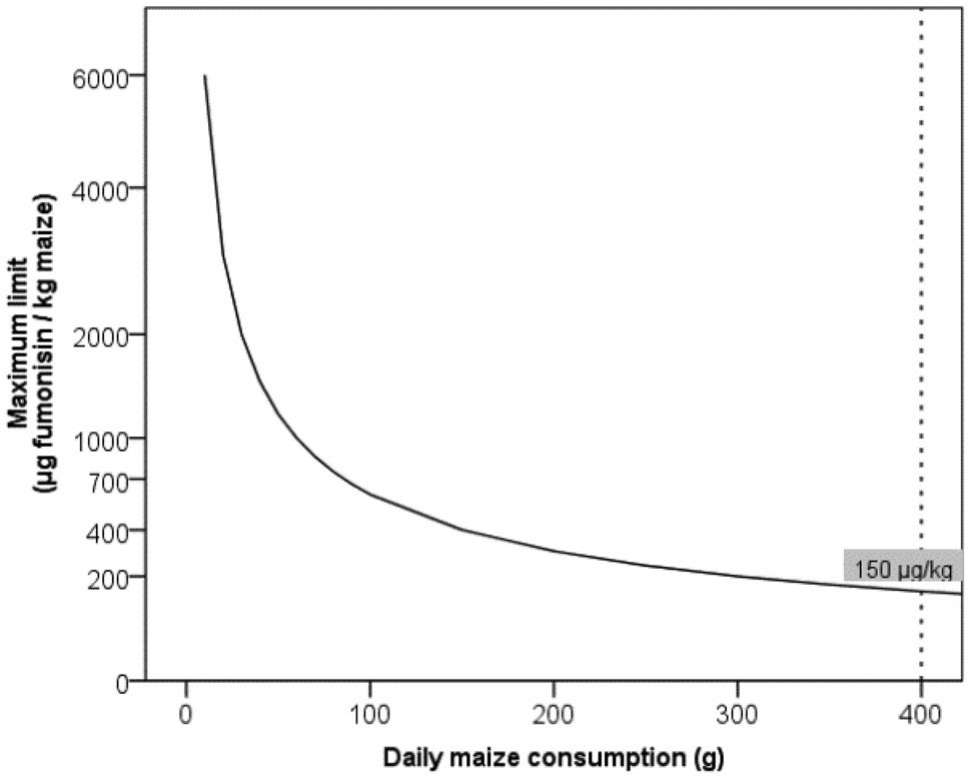
TDI curve for fumonisin for a 60-kg adult based on EFSA-guided group TDI for fumonisin (1.0 µg/kg bw/day). The area under each curve represents “safe area”; in contrast, the area above the curve represents the “unsafe area”. The shaded value (150 µg/kg) illustrate maximum limit that could be set for to protect a 60-kg adult with an average maize intake of 400 g (Matumba et al. 2019)

**Fig. 4:**
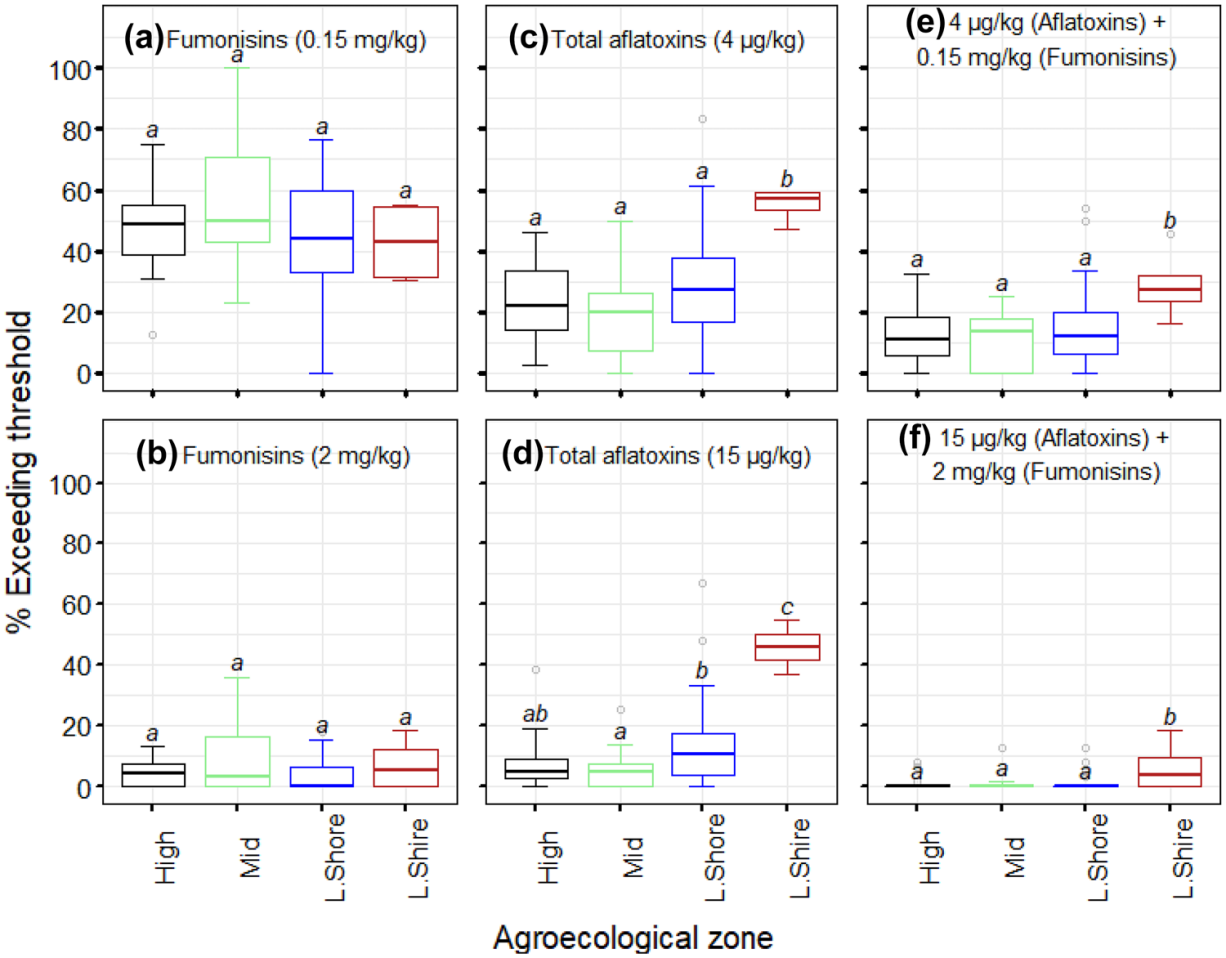
Summary of exceedance of aflatoxins and fumonisins limits in maize, as a proportion of all analysed samples in each Extension Planning Area of Malawi: **a** 0.15 mg/kg for fumonisins, **b** 2.0 mg/kg for fumonisins (MS 32: 2014), **c** 4 µg/kg for aflatoxins (EU regulatory limit), **d** 15 µg/kg for aflatoxins (MS 32:^2014^), **e** co-occurrence of fumonisins and aflatoxins above 0.15 mg/kg and 4 μg/kg, respectively, and **f** co-occurrence of fumonisins and aflatoxins above 2 mg/kg and 15 µg/kg, respectively. Agroecological zones with different letters have significant differences (*P* = 0.05). The dots, error bars, and upper and lower ends of the box represent outliers, spread, and first and third quartiles, respectively. Agroecological zones: highlands (high); mid-elevation (mid); Lake Shore and upper and middle Shire valley (L.Shore); lower Shire valley (L.Shire)

The Malawi standard on maize grain quality specifies 2 mg/kg of total fumonisins (MS 32:^2014^). The Codex Alimentarius has set the maximum limit for fumonisins in food at 4 mg/kg (Codex 2015), which is the same as the EU fumonisin regulation for unprocessed maize (EC 2007). However, the European Food Safety Authority (EFSA) Panel on Contaminants in the Food Chain (CONTAM) established a group tolerable daily intake (TDI) for fumonisins of 1.0 µg/kg body weight (bw) per day (EFSA 2018). Using this expert opinion, a 60-kg adult consumer with an average daily intake of about 400 g of maize as is the case in Malawi (Matumba et al. 2019) requires a maximum tolerable limit of 150 µg/kg of fumonisins to be adequately protected (Fig. 3). In general, almost all positive samples from all AEZ exceeded the proposed regulatory limit of 0.15 mg/kg but fell short of the EU regulatory limit of 4 mg/kg (Fig. 2b). The proportion of samples exceeding 0.15 and 2 mg/kg fumonisins did not vary significantly across the AEZs (Fig. 4a, b). The proposed limit of 0.15 mg/kg fumonisins in maize could adequately protect adult consumers, considering the frequency with which maize is consumed in Malawi. However, it may not effectively protect children, and its strict application could certainly have food security implications.

The Malawi standard for maize grain specifies 15 µg/kg for total aflatoxins and 5 µg/kg for AFB_1_ (MS 32: 2014). Out of the positive samples, 75.6%, 67.8%, and 51.1% of the samples exceeded the EU regulatory limit (4 µg/kg; EC 2010), the Malawi standard for maize of 15 µg/kg (MS 32:^2014^), and the US Food and Drug Administration’s (FDA 2000) limit for human food (20 µg/kg, total aflatoxin), respectively. In addition, as a proportion of all analysed samples, the lower Shire AEZ had a higher percentage of samples (*P* < 0.05) exceeding 4 and 15 µg/kg than the other AEZs (Fig. 4c, d). For the highlands, 38.8%, 9.0%, and 6.3% of the positive samples exceed 4, 15, and 20 µg/kg regulatory limits, respectively. In the Lake Shore and Upper and Middle Shire, 36.5%, 19.8%, and 15.0% of the positive samples exceed 4, 15, and 20 µg/kg regulatory limits, respectively, whereas for the mid-elevation, 24.6%, 12.5%, and 6.3% of the positive samples exceed 4, 15, and 20 µg/kg regulatory limits, respectively.

Aflatoxins and fumonisins were found to frequently co-occur in maize samples (Fig. 4e, f). In general, both aflatoxins and fumonisins were detected together in 38.6% of all maize samples, with co-occurrence of 41.2%, 36.2%, 37.6%, and 37.8% in the highlands, lower Shire, Lake Shore and upper and middle Shire, and mid-elevation AEZs. However, the lower Shire River valley AEZ had a higher (*P* < 0.05) co-occurrence of aflatoxins and fumonisins at levels exceeding aflatoxin regulatory limits of 4 and 15 µg/kg and fumonisin limit in maize products of 0.15 and 2 mg/kg (Fig. 4e, f).

### Total aflatoxins are more responsive to micro-climate variations than total fumonisins in maize

**Fig. 5:**
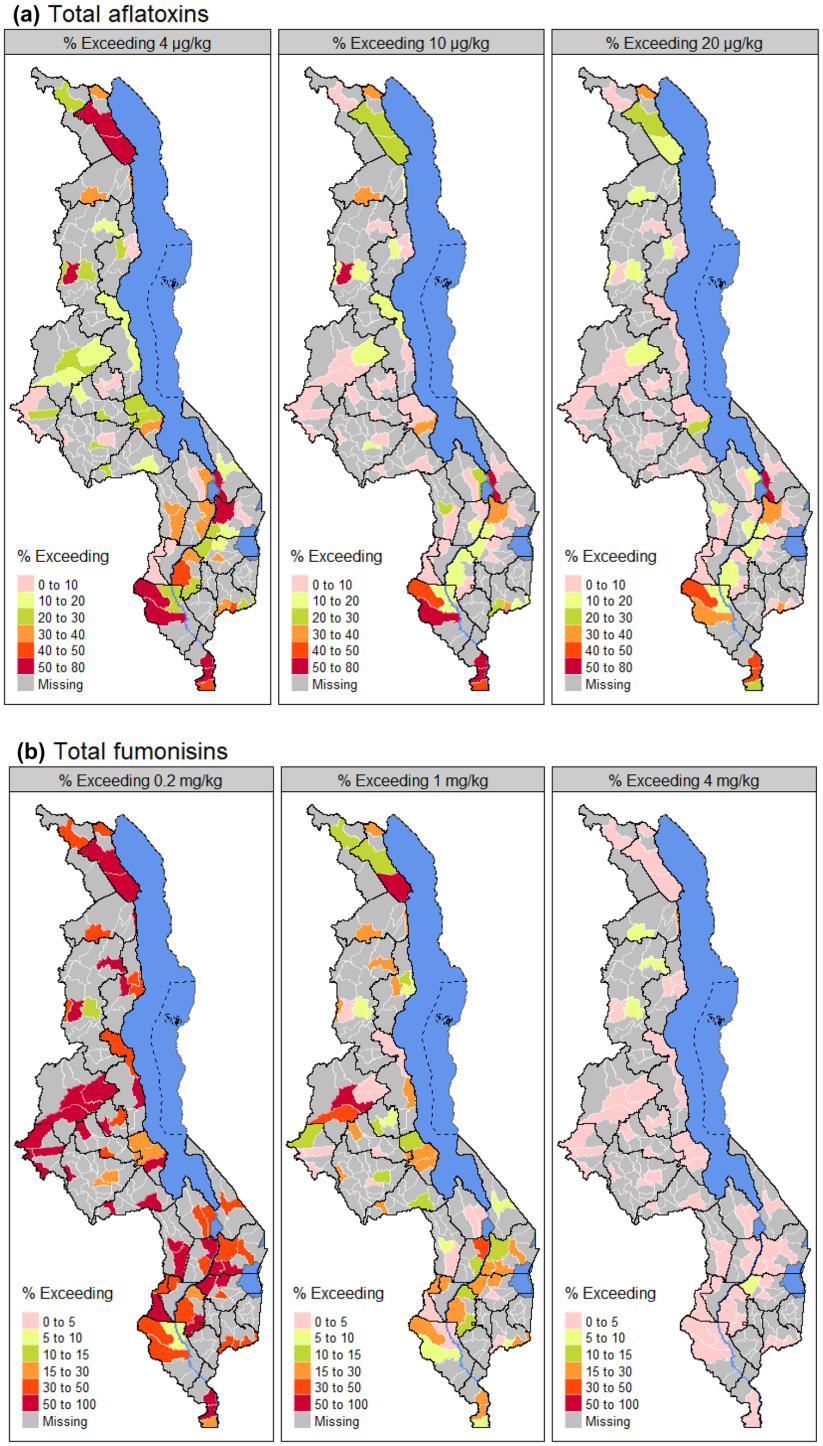
Spatial distribution of the proportion of all samples exceeding selected thresholds of total aflatoxins (upper panel) and total fumonisins (lower panel) in Extension Planning Areas

**Fig. 6:**
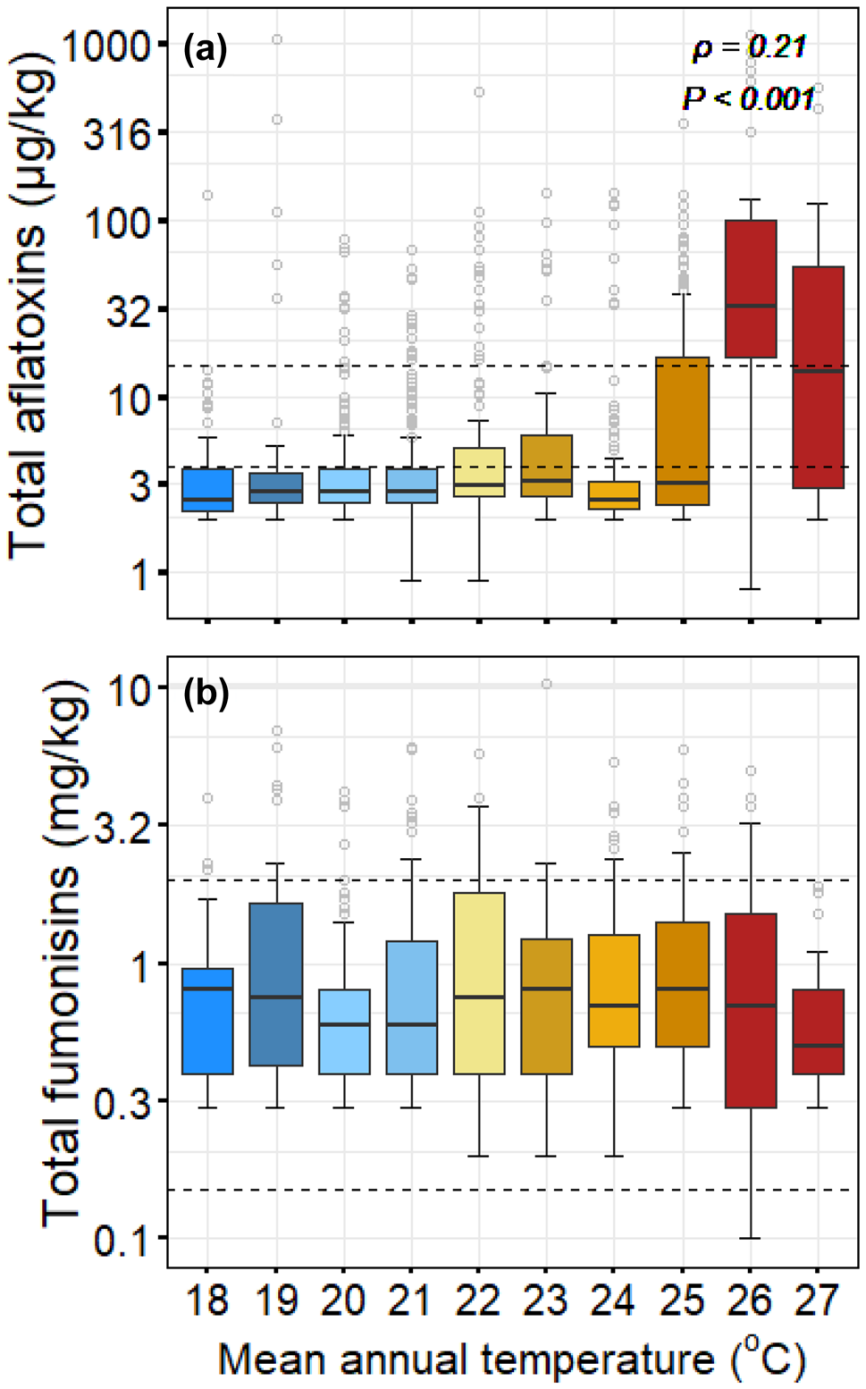
Boxplots of total aflatoxins and fumonisins grouped by mean annual temperature (based on Extension Planning Areas of Malawi). Dashed lines show guideline value (see text). Spearman’s rank correlation coefficient and *P* value shown for results in plot (**a**)

Several high (outlier) values for both total aflatoxins and total fumonisins are distributed across the range of climate settings sampled in this study (Fig. 2), and we did not find significant differences (*P* > 0.05) in contamination rate (% positive samples) of both total aflatoxins and total fumonisins across the AEZs. However, although exceedances of guideline values in levels of total aflatoxin can be observed in all AEZs, overall lower counts are found in the highlands and mid-elevation AEZs (Fig. 4). These AEZs are associated with relatively lower annual mean temperature compared to the Lower Shire and Lake Shore AEZs (Figs. 1 and 2). Figure 5 shows the spatial variation of the proportion of all samples exceeding selected thresholds of total aflatoxins and total fumonisins in each sampled Extension Planning Area (EPA). Significantly higher exceedances of thresholds for total aflatoxins are associated with sites characterised by higher mean annual temperature. Most of the EPAs with high proportion of samples with levels of total aflatoxins exceeding 4 µg/kg were in the lower Shire and the Lake Shore AEZs. However, the EPAs in the lower Shire AEZ show a relatively higher proportion of samples with levels of total aflatoxins exceeding 10 and µg/kg than those in the Lake Shore AEZ (Fig. 5). In addition, there are significantly higher exceedance rates at locations with higher annual average temperature (*ρ* = 0.21, *P* < 0.05, Spearman’s rank correlation), and the relationship is particularly clear for sites where the annual mean temperature is 24 °C (Fig. 6). Therefore, the wide variation in the levels of total aflatoxins in maize samples across the AEZs reflects the micro-climatic differences of the AEZs (Jaime-Garcia and Cotty 2010). However, levels of total fumonisins in maize did not show the same clear zonation as total aflatoxins. Exceedances for total fumonisins were not significantly different across the AEZs (Figs. 5 and 6). Therefore, results from this study suggest that levels of total aflatoxins in maize are more responsive to climatic variables such as temperature than levels of total fumonisins.

A similar geographical pattern, as in this study, in the occurrence of total aflatoxins in Malawian maize samples was reported in earlier studies (Matumba et al. 2014c; Chipinga 2014; Mwalwayo and Thole 2016), albeit from a smaller sample size. A similar geographical pattern was also reflected in AFB_1_-lysine adduct levels in blood samples from Malawian rural population (Seetha et al. 2018). The influence of climatic variations on the geographical distribution and levels of fumonisins and aflatoxins has also been reported in the region (e.g., Mukanga et al. 2010; Rheeder et al. 2016; Hove et al. 2016) and globally (e.g., Shelby et al. 1994). Generally, relative humidity and temperature are the most critical climatic risk factors for *Aspergillus* *flavus* colonisation and aflatoxin production during drying and storage (Magan et al. 2003; Chauhan et al. 2016; Battilani et al. 2008, 2013). Aflatoxins are produced optimally at temperatures between 25 and 35 °C, depending on water activity (Achglinkame et al. 2017), which coincide with the diurnal temperature range for low-lying AEZs of the Lake Shore and lower Shire River valley (Fig. 1). The low-lying AEZs are associated with high air temperatures and erratic rainfall (Fig. 1) throughout the year (Met Malawi 2006). On the other hand, fumonisins are produced optimally under a relatively wider range of temperatures (15–30 °C) (Alberts et al. 1990; Murphy et al. 1993; Marín et al. 2004; Samapundo et al. 2005; Mogensen et al. 2009; Wu et al. 2011; Medina et al. 2013). Hence, temperatures in all the AEZs are within the range conducive for *Fusarium* spp. growth and fumonisin production. Therefore, despite significant variations in aflatoxins, there may be no significant differences across the AEZs in fumonisin levels.

As a staple food, contamination of maize with aflatoxins and fumonisins poses a public health risk in Malawi, which may be particularly serious for rural subsistence farming communities and children who may suffer from enhanced exposure (Seetha et al. 2018; Braun and Wink 2018). In Malawi, the risk is compounded by poorly diversified household food consumption (Matumba et al. 2016; Ambler et al. 2017). Further, the link between climatic conditions and the contamination (levels and distribution) of maize with aflatoxins and fumonisins means that this risk is likely to increase with climate change, unless mitigation measures are put in place. In Malawi, Ngongondo et al. (2015) reported a decreasing annual rainfall regime and increasing temperature (*P* < 0.05) between 1970 and 2001. The future climate is also expected to become drier (reduction of up to 4% in annual rainfall levels), with more erratic rainfall and temperature projected to rise by 1–2.5 °C (Warnatzsch et al. 2020). These changes are projected to make the conditions more conducive for contamination of maize with mycotoxins (Battilani et al. 2008, 2016; Paterson and Lima 2010; Magan et al. 2011; Warnatzsch et al. 2020).

In this regard, this study raises awareness of contamination of maize, in Malawi, with aflatoxins and fumonisins and highlights the priority areas of the country for interventions. Enforcing the proposed limit of 0.15 mg/kg fumonisins in maize could protect the adult maize consumers in Malawi. However, its strict application could certainly have food security implications. Food safety, with its emphasis on food quality, is frequently subordinate to issues of food security, with their emphasis on sufficiency of supply, because of chronic shortages of staple foods due to tenuous agricultural production systems. Although there are regulations limiting aflatoxins concentrations in food, on their own, they have limited impact as they cannot be strictly applied to the large population of subsistence farmers in Malawi (Matumba et al. 2014d). Therefore, it is imperative that holistic measures are put in place to improve agronomy, storage, handling, and regulation (Matumba et al. 2021), to control the levels of aflatoxins and fumonisins in maize at the point of consumption.

## Supplementary Information

Below is the link to the electronic supplementary material.

Supplementary file1 (DOCX 57 kb)

## Data Availability

All data generated and analysed during this study are included in this published article and its supplementary information files.
