# Correction to: Micro‑climatic variations across Malawi have a greater influence on contamination of maize with aflatoxins than with fumonisins

**DOI:** 10.1007/s12550-023-00482-6

**Published:** 2023-05-01

**Authors:** Justin Temwani Ng’ambi, Joseph Atehnkeng, Maurice Monjerezi, Cosmo Ngongondo, Ephraim Vunain, Connel Ching’anda, Alejandro Ortega‑Beltran, Peter J. Cotty, Limbikani Matumba, R. Bandyopadhyay

**Affiliations:** 1 Faculty of Science, University of Malawi, P.O. Box 280, Zomba, Malawi; 2 International Institute of Tropical Agriculture (IITA), Route Kavumu, Km 18, Bifurcation, Birava, Site UCB, PO Box 1222, Bukavu, Democratic Republic of the Congo; 3 School of Plant Sciences, University of Arizona, Tucson, AZ USA; 4 International Institute of Tropical Agriculture (IITA), Ibadan, Nigeria; 5 College of Food Science and Engineering, Ocean University of China, Qingdao, China; 6 USDA-ARS, Tucson, AZ USA; 7 Food Technology and Nutrition Group, Lilongwe University of Agriculture and Natural Resources (LUANAR), Natural Resources College, P.O. Box 143, Lilongwe, Malawi; 8 MYTOX‑SOUTH International Thematic Network, Ghent University, Ghent, Belgium

**Correction to: Mycotoxin Research** **https://doi.org/10.1007/s12550-022-00471-1**

The article “Micro‑climatic variations across Malawi have a greater influence on contamination of maize with aflatoxins than with fumonisins”, written by Ng’ambi, J.T., Atehnkeng, J., Monjerezi, M. et al., was originally published electronically on the publisher’s internet portal on 18 November 2022 without open access. With the author(s)’ decision to opt for Open Choice the copyright of the article changed on 14 April 2023 to © The Author(s) 2023 and the article is forthwith distributed under a Creative Commons Attribution 4.0 International License, which permits use, sharing, adaptation, distribution and reproduction in any medium or format, as long as you give appropriate credit to the original author(s) and the source, provide a link to the Creative Commons licence, and indicate if changes were made. The images or other third party material in this article are included in the article’s Creative Commons licence, unless indicated otherwise in a credit line to the material. If material is not included in the article’s Creative Commons licence and your intended use is not permitted by statutory regulation or exceeds the permitted use, you will need to obtain permission directly from the copyright holder. To view a copy of this licence, visit http://creativecommons.org/licenses/by/4.0.

The original article has been corrected.

