# Supplementary material for: Micro-climatic variations across Malawi have a greater influence on contamination of maize with aflatoxins than with fumonisins
Source: Mycotoxin Res. 2022 Nov 29;39(1):33–44. doi: 10.1007/s12550-022-00471-1 (PMC10156841; doi:10.1007/s12550-022-00471-1)
Supplement: Supplementary file 1 — Supplementary file1 (DOCX 57 kb) [file 12550_2022_471_MOESM1_ESM.docx]

**Supplementary information**

Table s1. Occurrence and comparison of total aflatoxins detected in the samples, percentage of positive samples (Pos), and mean ± standard error (SEM), minimum and maximum values in positive samples, median and percentage exceeding selected regulatory levels (for positive samples).

| District | EPA | *n* | Pos  (%) | Min  (µg/kg) | Max  (µg/kg) | Mean±SEM  (µg/kg) | Median  (µg/kg) | >4  µg/kg  (%) | >15  µg/kg  (%) |
| --- | --- | --- | --- | --- | --- | --- | --- | --- | --- |
| ***Highlands AEZ*** | | | | | | | | | |
| Dedza | Bembeke | 38 | 63.2 | 2.0 | 14.2 | 4.16±0.58 | 2.60 | 13.2 | 0.0 |
| Dedza | Chafumbwa | 40 | 75.0 | 2.0 | 138.7 | 8.24±3.92 | 2.65 | 20.0 | 2.5 |
| Ntchisi | Chikwapula | 26 | 84.6 | 2.1 | 5.1 | 2.74±0.13 | 2.60 | 3.8 | 0.0 |
| Ntchisi | Chipuka | 37 | 81.1 | 2.1 | 31.3 | 3.71±0.86 | 2.70 | 2.7 | 2.7 |
| Dowa | Chivala | 48 | 93.8 | 2.0 | 76.8 | 5.70±1.73 | 3.20 | 20.8 | 4.2 |
| Mzimba | Kazomba | 16 | 81.3 | 2.1 | 47.5 | 8.29±3.21 | 3.30 | 25.0 | 12.5 |
| Chitipa | Lufita | 35 | 100 | 2.6 | 47.6 | 5.97±1.62 | 3.40 | 22.9 | 5.7 |
| Dowa | Madisi | 8 | 87.5 | 2.4 | 4.2 | 3.39±0.23 | 3.40 | 12.5 | 0.0 |
| Ntcheu | Manjawila | 37 | 86.5 | 2.1 | 15.6 | 4.87±0.52 | 3.80 | 37.8 | 2.7 |
| Mulanje | Milonde | 39 | 76.9 | 2.1 | 110.1 | 11.84±4.17 | 3.00 | 23.1 | 7.7 |
| Mulanje | Msikawanjala | 11 | 90.9 | 2.3 | 19.0 | 6.42±1.64 | 3.65 | 36.4 | 9.1 |
| Mulanje | Mulanje Boma | 13 | 84.6 | 2.1 | 67.6 | 18.00±5.87 | 6.00 | 46.2 | 38.5 |
| Mzuzu | Mzuzu City | 54 | 77.8 | 2.0 | 1072.0 | 41.98±23.59 | 2.90 | 16.7 | 9.3 |
| Ntcheu | Nsipe | 21 | 100 | 2.0 | 69.5 | 13.26±4.45 | 3.90 | 38.1 | 19.0 |
| ***Lake Shore, Middle and Upper Shire AEZ*** | | | | | | | | | |
| Zomba | Chingale | 20 | 70.0 | 2.3 | 53.1 | 9.34±3.21 | 3.30 | 25.0 | 10.0 |
| Salima | Chinguluwe | 4 | 50.0 | 2.7 | 4.3 | 3.50±0.57 | 3.50 | 25.0 | 0.0 |
| Rumphi | Chiweta | 20 | 90.0 | 2.0 | 55.1 | 9.77±3.52 | 2.80 | 35.0 | 15.0 |
| Chikwawa | Kalambo | 21 | 76.2 | 2.1 | 121.2 | 43.42±9.21 | 33.40 | 52.4 | 47.6 |
| Salima | Khombedza | 17 | 64.7 | 2.0 | 59.0 | 9.10±4.04 | 3.70 | 29.4 | 5.9 |
| Blantyre | Kunthembwe | 9 | 88.9 | 2.6 | 56.9 | 10.98±6.23 | 3.90 | 44.4 | 11.1 |
| Nkhatabay | Limphasa | 41 | 61.0 | 2.1 | 40.6 | 4.54±1.21 | 2.50 | 7.3 | 2.4 |
| Nkhotakota | Linga | 51 | 82.4 | 2.0 | 122.8 | 6.97±2.66 | 2.80 | 13.7 | 3.9 |
| Blantyre | Lirangwe | 26 | 88.5 | 2.0 | 53.1 | 7.83±2.39 | 3.30 | 30.8 | 7.7 |
| Karonga | Lupembe | 8 | 100.0 | 2.3 | 39.7 | 12.84±5.68 | 4.85 | 50.0 | 25.0 |
| Mangochi | Maiwa | 6 | 83.3 | 11.1 | 44.1 | 24.00±5.00 | 21.00 | 83.3 | 66.7 |
| Zomba | Malosa | 17 | 70.6 | 2.1 | 8.3 | 3.50±0.47 | 2.75 | 17.6 | 0.0 |
| Chikwawa | Mitole | 13 | 46.2 | 2.0 | 121.5 | 38.20±15.10 | 4.95 | 23.1 | 15.4 |
| Nkhatabay | Mpamba | 10 | 60.0 | 2.1 | 2.7 | 2.33±0.09 | 2.20 | 0.0 | 0.0 |
| Karonga | Mpata | 13 | 100.0 | 2.4 | 136.9 | 17.97±10.22 | 5.20 | 61.5 | 23.1 |
| Balaka | Mpilisi | 34 | 82.4 | 2.0 | 141.0 | 16.06±5.65 | 3.30 | 38.2 | 11.8 |
| Mangochi | Mthiramanja | 7 | 71.4 | 2.0 | 2.4 | 2.24±0.08 | 2.40 | 0.0 | 0.0 |
| Machinga | Mtubwi | 24 | 87.5 | 2.1 | 141.2 | 13.86±6.62 | 2.50 | 25.0 | 12.5 |
| Mwanza | Mwanza | 5 | 40.0 | 2.3 | 3.8 | 3.05±0.47 | 3.05 | 0.0 | 0.0 |
| Nkhatabay | Mzenga | 9 | 66.7 | 2.3 | 33.2 | 8.50±4.08 | 2.90 | 22.2 | 11.1 |
| Machinga | Mbonechera | 10 | 100.0 | 2.4 | 69.3 | 19.26±8.30 | 4.20 | 50.0 | 30.0 |
| Mangochi | Nasenga | 9 | 66.7 | 2.1 | 78.4 | 16.82±10.12 | 3.65 | 33.3 | 11.1 |
| Nkhotakota | Nkhunga | 11 | 90.9 | 2.0 | 24.3 | 5.94±2.11 | 3.25 | 18.2 | 9.1 |
| Mangochi | Ntiya | 15 | 53.3 | 2.1 | 17.3 | 4.70±1.35 | 2.50 | 13.3 | 6.7 |
| Karonga | Nyungwe | 9 | 100 | 2.1 | 59.8 | 13.80±6.58 | 2.80 | 33.3 | 33.3 |
| Salima | Tembwe | 16 | 75.0 | 2.2 | 358.0 | 43.76±25.12 | 5.25 | 37.5 | 31.3 |
| MWANZA | Thambani | 22 | 36.4 | 2.0 | 17.9 | 6.39±1.48 | 2.85 | 9.1 | 9.1 |
| Balaka | Ulongwe | 6 | 100 | 2.2 | 6.7 | 3.83±0.76 | 3.10 | 33.3 | 0.0 |
| ***Lower Shire AEZ*** | | | | | | | | | |
| Chikwawa | Mbewe | 56 | 91.1 | 2.0 | 566.0 | 45.20±13.20 | 13.60 | 58.9 | 42.9 |
| Nsanje | Mpatsa | 22 | 68.2 | 2.0 | 130.3 | 47.23±8.72 | 33.70 | 59.1 | 54.5 |
| Nsanje | Nyachilenda | 19 | 52.6 | 2.1 | 64.0 | 24.08±4.28 | 19.60 | 47.4 | 36.8 |
| Nsanje | Zunde | 29 | 58.6 | 2.2 | 1122.0 | 341.35±73.67 | 92.30 | 55.2 | 48.3 |
| ***Mid elevation AEZ*** | | | | | | | | | |
| Rumphi | Bolero | 16 | 81.3 | 2.1 | 540.0 | 55.84±36.85 | 2.70 | 37.5 | 25.0 |
| Lilongwe | Chileka | 22 | 59.1 | 2.1 | 23.5 | 5.77±1.55 | 2.80 | 9.1 | 9.1 |
| Kasungu | Chipala | 22 | 100.0 | 2.0 | 33.6 | 4.64±1.40 | 3.00 | 22.7 | 4.5 |
| Lilongwe | Chitsime | 65 | 76.9 | 2.0 | 28.5 | 5.40±0.67 | 2.85 | 23.1 | 6.2 |
| Kasungu | Lisasadzi | 10 | 90.0 | 2.0 | 5.8 | 3.17±0.36 | 2.90 | 10.0 | 0.0 |
| Mzimba | Manyamula | 2 | 100.0 | 2.7 | 12.1 | 7.40±4.70 | 7.40 | 50.0 | 0.0 |
| Mchinji | Mikundi | 4 | 100 | 2.0 | 7.0 | 3.60±1.15 | 2.70 | 25.0 | 0.0 |
| Mzimba | Mjinge | 22 | 86.4 | 2.1 | 67.7 | 10.22±3.73 | 2.60 | 27.3 | 13.6 |
| Mchinji | Mkanda | 24 | 83.3 | 2.0 | 5.5 | 2.86±0.16 | 2.80 | 4.2 | 0.0 |
| Mchinji | Mlonyeni | 7 | 71.4 | 2.0 | 3.1 | 2.16±0.31 | 2.30 | 0.0 | 0.0 |
| Lilongwe | Mpenu | 17 | 76.5 | 2.0 | 4.6 | 2.80±0.18 | 2.60 | 5.9 | 0.0 |
| Machinga | Nanyumbu | 7 | 100 | 2.3 | 3.5 | 2.93±0.15 | 2.90 | 0.0 | 0.0 |
| Blantyre | Ntonda | 35 | 88.6 | 2.1 | 26.1 | 4.49±0.81 | 3.00 | 20.0 | 5.7 |
| Zomba | Thondwe | 20 | 90.0 | 2.2 | 24.0 | 4.82±1.14 | 3.00 | 30.0 | 5.0 |
| Kasungu | Wimbe | 10 | 80.0 | 2.6 | 110.4 | 16.56±11.99 | 3.10 | 10.0 | 10.0 |

Table s2. Occurrence and comparison of total fumonisins detected in the samples, percentage of positive samples (Pos), and mean ± standard error (SEM), minimum and maximum values in positive samples, median and percentage exceeding selected regulatory levels (for positive samples).

| District | EPA | *n* | Pos (%) | Min  (mg/kg) | Max  (mg/kg) | Mean ± SEM (mg/kg) | Median (mg/kg) | >0.15 mg/kg (%) | >2 mg/kg  (%) |
| --- | --- | --- | --- | --- | --- | --- | --- | --- | --- |
| ***Highlands AEZ*** | | | | | | | | | |
| Dedza | Bembeke | 38 | 52.63 | 0.30 | 4.00 | 0.96±0.14 | 0.80 | 52.63 | 5.26 |
| Dedza | Chafumbwa | 40 | 67.50 | 0.30 | 4.00 | 0.88±0.13 | 0.80 | 67.50 | 5.00 |
| Ntchisi | Chikwapula | 26 | 30.77 | 0.30 | 4.20 | 1.09±0.26 | 0.60 | 30.77 | 3.85 |
| Ntchisi | Chipuka | 37 | 51.35 | 0.30 | 1.80 | 0.79±0.07 | 0.60 | 51.35 | 0.00 |
| Dowa | Chivala | 48 | 35.42 | 0.30 | 1.40 | 0.59±0.04 | 0.50 | 35.42 | 0.00 |
| Mzimba | Kazomba | 16 | 12.50 | 0.40 | 6.10 | 3.25±1.01 | 3.25 | 12.50 | 6.25 |
| Chitipa | Lufita | 35 | 40.00 | 0.30 | 2.10 | 0.91±0.11 | 0.65 | 40.00 | 2.86 |
| Dowa | Madisi | 8 | 75.00 | 0.40 | 3.90 | 1.23±0.48 | 0.65 | 75.00 | 12.50 |
| Ntcheu | Manjawila | 37 | 51.35 | 0.30 | 1.50 | 0.53±0.05 | 0.40 | 51.35 | 0.00 |
| Mulanje | Milonde | 39 | 38.46 | 0.30 | 4.00 | 1.49±0.21 | 1.10 | 38.46 | 10.26 |
| Mulanje | Msikawanjala | 11 | 45.45 | 0.30 | 0.90 | 0.64±0.07 | 0.60 | 45.45 | 0.00 |
| Mulanje | Mulanje Boma | 13 | 46.15 | 0.40 | 2.70 | 1.03±0.23 | 0.80 | 46.15 | 7.69 |
| Mzuzu | Mzuzu City | 54 | 55.56 | 0.30 | 7.00 | 1.57±0.24 | 0.75 | 55.56 | 12.96 |
| Ntcheu | Nsipe | 21 | 57.14 | 0.40 | 1.70 | 0.73±0.08 | 0.65 | 57.14 | 0.00 |
| ***Lake Shore, Middle and Upper Shire AEZ*** | | | | | | | | | |
| Zomba | Chingale | 20 | 65.00 | 0.40 | 6.00 | 1.55±0.35 | 0.90 | 65.00 | 15.00 |
| Salima | Chinguluwe | 4 | 25.00 | 1.30 | 1.30 | 1.30±0.33 | 1.30 | 25.00 | 0.00 |
| Rumphi | Chiweta | 20 | 60.00 | 0.30 | 6.00 | 1.98±0.49 | 0.90 | 60.00 | 15.00 |
| Chikwawa | Kalambo | 21 | 42.86 | 0.30 | 2.00 | 0.96±0.13 | 1.10 | 42.86 | 0.00 |
| Salima | Khombedza | 17 | 17.65 | 0.30 | 4.00 | 1.83±0.47 | 1.20 | 17.65 | 5.88 |
| Blantyre | Kunthembwe | 9 | 33.33 | 0.30 | 1.20 | 0.87±0.16 | 1.10 | 33.33 | 0.00 |
| Nkhatabay | Limphasa | 41 | 48.78 | 0.20 | 2.60 | 0.84±0.10 | 0.65 | 48.78 | 4.88 |
| Nkhotakota | Linga | 51 | 76.47 | 0.30 | 5.40 | 1.11±0.14 | 0.70 | 76.47 | 7.84 |
| Blantyre | Lirangwe | 26 | 23.08 | 0.20 | 1.80 | 0.97±0.12 | 1.00 | 23.08 | 0.00 |
| Karonga | Lupembe | 8 | 75.00 | 0.50 | 2.50 | 0.95±0.27 | 0.65 | 75.00 | 12.50 |
| Mangochi | Maiwa | 6 | 0.00 | 0.00 | 0.00 | 0.00 | 0.00 | 0.00 | 0.00 |
| Zomba | Malosa | 17 | 58.82 | 0.30 | 3.50 | 1.44±0.27 | 0.95 | 58.82 | 17.65 |
| Chikwawa | Mitole | 13 | 7.69 | 0.70 | 0.70 | 0.70±0.05 | 0.70 | 7.69 | 0.00 |
| Nkhatabay | Mpamba | 10 | 40.00 | 0.30 | 1.40 | 0.68±0.16 | 0.50 | 40.00 | 0.00 |
| Karonga | Mpata | 13 | 69.23 | 0.60 | 3.70 | 1.63±0.26 | 1.40 | 69.23 | 15.38 |
| Balaka | Mpilisi | 34 | 32.35 | 0.40 | 10.30 | 1.74±0.50 | 0.70 | 32.35 | 5.88 |
| Mangochi | Mthiramanja | 7 | 42.86 | 0.40 | 0.50 | 0.47±0.02 | 0.50 | 42.86 | 0.00 |
| Machinga | Mtubwi | 24 | 62.50 | 0.30 | 2.30 | 0.88±0.14 | 0.50 | 62.50 | 4.17 |
| Mwanza | Mwanza | 5 | 60.00 | 0.50 | 1.00 | 0.70±0.12 | 0.60 | 60.00 | 0.00 |
| Nkhatabay | Mzenga | 9 | 66.67 | 0.30 | 3.70 | 1.18±0.46 | 0.45 | 66.67 | 11.11 |
| Machinga | Mbonechera | 10 | 30.00 | 0.40 | 1.10 | 0.73±0.11 | 0.70 | 30.00 | 0.00 |
| Mangochi | Nasenga | 9 | 33.33 | 0.50 | 0.70 | 0.57±0.04 | 0.50 | 33.33 | 0.00 |
| Nkhotakota | Nkhunga | 11 | 45.45 | 0.30 | 0.90 | 0.56±0.07 | 0.60 | 45.45 | 0.00 |
| Mangochi | Ntiya | 14 | 42.86 | 0.30 | 1.10 | 0.67±0.09 | 0.60 | 42.86 | 0.00 |
| Karonga | Nyungwe | 9 | 33.33 | 0.30 | 1.80 | 1.23±0.27 | 1.60 | 33.33 | 0.00 |
| Salima | Tembwe | 16 | 50.00 | 0.40 | 2.00 | 0.95±0.15 | 0.70 | 50.00 | 0.00 |
| MWANZA | Thambani | 22 | 45.45 | 0.30 | 3.00 | 1.11±0.18 | 0.85 | 45.45 | 4.55 |
| Balaka | Ulongwe | 6 | 50.00 | 0.50 | 2.00 | 1.20±0.31 | 1.10 | 50.00 | 0.00 |
| ***Lower Shire AEZ*** | | | | | | | | | |
| Chikwawa | Mbewe | 56 | 30.36 | 0.30 | 1.90 | 0.75±0.07 | 0.50 | 30.36 | 0.00 |
| Nsanje | Mpatsa | 22 | 54.55 | 0.30 | 4.00 | 1.60±0.28 | 1.10 | 54.55 | 18.18 |
| Nsanje | Nyachilenda | 19 | 36.84 | 0.30 | 1.60 | 0.61±0.12 | 0.50 | 31.58 | 0.00 |
| Nsanje | Zunde | 29 | 55.17 | 0.30 | 5.00 | 1.21±0.27 | 0.50 | 55.17 | 10.34 |
| ***Mid elevation AEZ*** | | | | | | | | | |
| Rumphi | Bolero | 16 | 43.75 | 0.50 | 5.80 | 1.94±0.48 | 0.90 | 43.75 | 18.75 |
| Lilongwe | Chileka | 22 | 54.55 | 0.40 | 3.70 | 1.49±0.22 | 1.05 | 54.55 | 13.64 |
| Kasungu | Chipala | 14 | 100.00 | 0.30 | 2.90 | 1.39±0.26 | 1.00 | 100.00 | 35.71 |
| Lilongwe | Chitsime | 65 | 23.08 | 0.30 | 3.90 | 0.95±0.14 | 0.40 | 23.08 | 3.08 |
| Kasungu | Lisasadzi | 10 | 70.00 | 0.40 | 1.60 | 0.93±0.14 | 0.80 | 70.00 | 0.00 |
| Mzimba | Manyamula | 2 | 50.00 | 1.00 | 1.00 | 1.00±0.50 | 1.00 | 50.00 | 0.00 |
| Mchinji | Mikundi | 4 | 100.00 | 0.30 | 2.00 | 0.93±0.39 | 0.70 | 100.00 | 0.00 |
| Mzimba | Mjinge | 21 | 42.86 | 0.30 | 3.50 | 1.68±0.25 | 1.70 | 42.86 | 19.05 |
| Mchinji | Mkanda | 24 | 50.00 | 0.40 | 2.20 | 0.80±0.12 | 0.50 | 50.00 | 4.17 |
| Mchinji | Mlonyeni | 7 | 71.43 | 0.30 | 0.70 | 0.46±0.06 | 0.50 | 71.43 | 0.00 |
| Lilongwe | Mpenu | 17 | 29.41 | 0.30 | 1.80 | 0.90±0.17 | 0.50 | 29.41 | 0.00 |
| Machinga | Nanyumbu | 7 | 42.86 | 0.40 | 3.90 | 2.57±0.72 | 3.40 | 42.86 | 28.57 |
| Blantyre | Ntonda | 35 | 71.43 | 0.30 | 2.40 | 0.83±0.10 | 0.70 | 71.43 | 5.71 |
| Zomba | Thondwe | 20 | 35.00 | 0.30 | 0.60 | 0.47±0.02 | 0.50 | 35.00 | 0.00 |
| Kasungu | Wimbe | 10 | 70.00 | 0.30 | 0.50 | 0.39±0.03 | 0.40 | 70.00 | 0.00 |
